# A Gaussian Beam Based Recursive Stiffness Matrix Model to Simulate Ultrasonic Array Signals from Multi-Layered Media

**DOI:** 10.3390/s20164371

**Published:** 2020-08-05

**Authors:** Chirag Anand, Roger Groves, Rinze Benedictus

**Affiliations:** 1 Structural Integrity and Composites Group, Faculty of Aerospace Engineering, Delft University of Technology, 2629 HS Delft, The Netherlands; r.m.groves@tudelft.nl (R.G.); R.Benedictus@tudelft.nl (R.B.); 2 Aerospace Non-Destructive Testing Laboratory, Faculty of Aerospace Engineering, Delft University of Technology, 2629 HS Delft, The Netherlands

**Keywords:** Gaussian beam, full matrix capture, angular spectrum, recursive stiffness matrix, CFRP, modeling

## Abstract

Ultrasonic testing using arrays is becoming widely used to test composite structures in the Aerospace industry. In recent years, the Full Matrix Capture (FMC) technique has been implemented to extract the signals for post-processing to form an image. The inherent anisotropy and the layering of the structure pose challenges for the interpretation of this FMC data. To overcome this challenge, modeling techniques are required that take into account the diffraction caused by finite-size transducers and the response of the structure to these bounded beams. Existing models either homogenize the entire structure, use computationally expensive finite difference time domain (FDTD) methods, or do not consider the shape of the bounded beam, which is used to test such structures. This paper proposes a modeling technique based on combining the Multi-Gaussian beam model with the recursive stiffness matrix method to simulate the FMC signals for layered anisotropic media. The paper provides the steps required for the modeling technique, the extraction of the system efficiency factor, and validation of the model with experimentally determined signals for aluminum as an isotropic material such as aluminum and Carbon Fiber Reinforced Plastic (CFRP) laminate as a layered material. The proposed method is computationally inexpensive, shows good agreement with the experimentally determined FMC data, and enables us to understand the effects of various transducer and material parameters on the extracted FMC signals.

## 1. Introduction

In recent years, the use of composite materials in aerospace structures has risen significantly [1,2]. These structures mainly consist of multi-layered composite laminates. This has led to an increase in ultrasonic phased array testing of such structures [3]. The testing of such multi-layered structures is complicated due to the multiple reflections at the layer boundaries and the direction-dependent velocity caused due to the inherent anisotropy of such structures [4]. There is a need for computational models to simulate, analyze, and study the interaction of finite beam transducers with such multi-layered composite materials, taking into account the effects of multiple reflections and direction-dependent velocity.

A variety of approaches to simulate the response from multilayer structures have been reported in literature. One of these approaches is applying ray methods to the homogenized multi-layer structure [5]. Though these have the advantage of being applicable to planar and curved geometries, they fail to show the reflection and reverberation from the plies due to the homogenization of the material properties [5]. A hybrid ray-finite difference time domain (FDTD) approach was developed, which utilizes ray methods for propagation through the coupling medium and FDTD method to calculate the response of the layered medium [6]. This method can solve complex geometries but is time-consuming and computationally expensive. An alternative technique using multi-Gaussian beams to model the transducer radiation was proposed by Huang et al. for isotropic materials [7]. This was extended for phased array radiation through anisotropic media [8]. This method can be used for planar or nonplanar composites but is computationally very expensive due to the number of beams that need to be traced in a multi-layered media. 

A different approach is to use highly effective plane wave models from geophysics literature [9,10,11,12] to calculate the reflection and transmission at the layer interfaces. The Transfer matrix method developed by Thomson [11] and Haskell [10] are the basic matrix formulations that have been used. These suffer from instability problems for large frequency/thickness (fd) products where *f* is the frequency and *d* is the thickness of the layer [9]. Knopoff [9] suggested a global matrix method to overcome the instability problems. For anisotropic media, Nayfeh developed the transfer matrix method [13]. Rokhlin and Wang [14] developed recursive stiffness matrix method that uses individual layer stiffness matrices to build up the global stiffness matrix for the entire laminate.

The plane wave models for multi-layered media above do not reflect the real situation where a finite size transducer is used that emits a bounded beam. The diffraction of the bounded beam can be modeled using the angular spectrum, where the fields anywhere in the space can be defined as a sum of an infinite number of plane waves in different directions [15,16]. 

To simulate the signals received during testing of a material, it is important to model the beam emitted from the transducer elements. The transducer elements act as piston transducers. Piston transducers can be approximated as Gaussian beam transducers [17]. The directivity of such transducers can then be expressed as Gaussian beams. Gaussian beams are also nonsingular when interacting with curved surfaces, unlike plane waves. It was also shown by Huang et al. that phased array transducers can be modeled using multi-Gaussian beams [7]. Hence, to simulate the signals obtained while testing a structure, it is necessary to develop a model that is computationally inexpensive and couples the diffraction effect of the beam from the rectangular transducer element with the response of the structure under consideration. Such a model can then be used for testing and developing imaging algorithms, optimizing transducer parameters, and testing conditions.

Hence, this paper modeling technique is developed to carry out transducer array inspection of planar composite materials with the Full Matrix Capture (FMC) data acquisition. The novelty of the paper lies in the development of an analytical model based on the angular decomposition of multi-Gaussian beams combined with the response of the laminate to simulate the full matrix capture (FMC) signals. This model uses multi-Gaussian beams to represent the source beam combined with an experimentally determined system function and the total reflection coefficient of the material under inspection calculated using the recursive stiffness matrix method. 

Section 2 contains a brief explanation of the theory involved in the proposed modeling technique. Section 3 contains the application of the modeling techniques to aluminum and Carbon Fiber Reinforced Plastic (CFRP) laminate using arrays of different frequencies, element numbers, and sizes. Section 4 gives the conclusion of the paper.

## 2. Theory

### 2.1. Stiffness Matrix Method for Multi-Layer Wave Propagation

**Figure 1:**
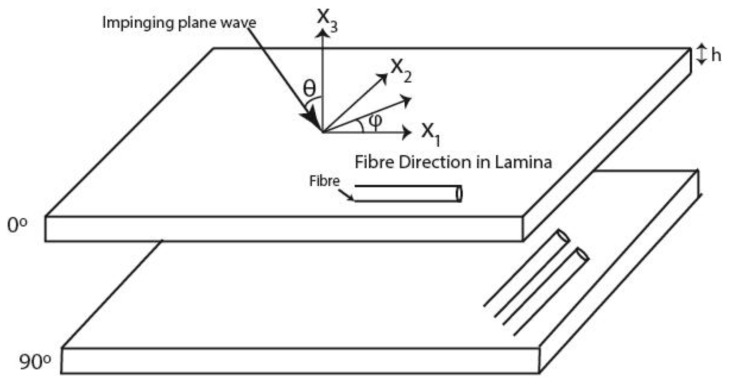
Carbon fiber reinforced plastic (CFRP) laminas specifying the local axis.

Let us consider a plane wave impinging on the top layer of a planar multi-layer laminate in the *x*_1_-*x*_3_ plane where the laminate consists of *n* number of layers as shown in Figure 1. 

These layers are assumed homogeneous and anisotropic. The anisotropic layers are assumed of infinite extent in the plane (*x*_1_-*x*_2_) normal to the thickness direction. The laminate is bounded by two semi-infinite bounding layers that are denoted by 0 and *n* + 1. The plane wave traveling from the upper bounding layer has an incident angle θ with respect to the *x*_3_ axis and its wave vector projection on the *x*_1_-*x*_2_ plane is denoted by φ. The plane wave displacement *u* in a layer is given by the Equation (1)

(1) u=exp(i(kx−ωt))

where *i* is the imaginary number, **k** is the wavenumber vector, *ω* is the angular frequency, and *t* is the time. Due to the application of Snell’s law [12], the wavenumber components in the plane of the interfaces should be equal throughout the laminate, i.e., *k*_1_ and *k*_2_ remain the same. The wavenumber component *k*_3_ can be calculated using the Christoffel equation as given below [13]:

(2) $$\left(c_{ijkl}k_{j}k_{k}-\rho \omega^{2}\partial_{il}\right)d_{l}=0$$

where *c_ijkl_* is the stiffness tensor, *ρ* is the density of the material, *δ_il_* is the Kronecker delta, *d*_l_ is the polarization vector component for different wave modes, and *i, j, k, l* consist of values 1, 2, 3, corresponding to the three axes *x*_1_*, x*_2_, *x*_3_. Equation (2) can be solved to obtain the values of the wavenumber component *k_3_*. *k*_3_ will have two solutions for each propagating wave mode. One solution corresponds to the downward going wave in the layer and the other corresponds to the upward traveling wave. The downward traveling wave is denoted by ‘+’ and the upward traveling wave in the layer by ‘-’. The wave modes are represented by *p* with values 1, 2, and 3. The quasi-longitudinal wave is represented by *p* = 3 and the quasi-shear waves are represented by *p* = 1, 2.

Hence, the displacement in the layer *m* is given below [14]:

(3) $$u_{i}^{m}=\sum_{p=1}^{3}\left(a^{m,p+}d_{i}^{m,p+}e^{ik_{3}^{m,p}(x_{3}-x_{3}^{m})}+a^{m,p-}d_{i}^{m,p-}e^{-ik_{3}^{m,p}(x_{3}-x_{3}^{m})}\right)e^{i\left(k_{1}x_{1}+k_{2}x_{2}-\omega t\right)}$$

where $a^{m,p+/-}$are the wave amplitudes of the downward and upward traveling waves of mode *p* in the layer *m*, and $d_{i}^{m,p+/-}$ is the *i*th component of the polarization vector of wave mode *p* in the layer *m*. The coordinates $x_{3}^{m}$ is the local coordinate of the layer m. The relationship between the stress and displacement in the layer is given below:

(4) $$\sigma_{ij}=\frac{1}{2}c_{ijkl}\left(\frac{\partial u_{k}}{\partial x_{l}}-\frac{\partial u_{l}}{\partial x_{k}}\right)$$

By substituting Equation (3) into Equation (4), a layer stiffness matrix *S_m_* is defined that relates the stresses and displacements at the top and bottom of the layer.

(5) $$\left[\begin{array}{l} \sigma_{1i}^{m}\left(0\right)\\ \sigma_{1i}^{m}\left(h_{m}\right) \end{array}\right]=S_{m}\left[\begin{array}{l} u_{i}^{m}\left(0\right)\\ u_{i}^{m}\left(h_{m}\right) \end{array}\right]$$

where $\sigma_{1i}^{m}\left(0\right)$, $\sigma_{1i}^{m}\left(h_{m}\right)$ are the stress components at the top and bottom of layer *m,* respectively, and $u_{i}^{m}\left(0\right)$, $u_{i}^{m}\left(h_{m}\right)$ are the displacement components at the top and bottom of layer *m,* respectively. In order to define the stiffness matrix for the entire structure, continuity of stress and displacement is applied at each interface. The equation relating the stress in the upper semi-infinite bounding layer and the lower semi-infinite bounding layer is given below:

(6) $$\left[\begin{array}{l} \sigma_{1i}^{0}\\ \sigma_{1i}^{n+1} \end{array}\right]=S_{N}\left[\begin{array}{l} u_{i}^{0}\left(0\right)\\ u_{i}^{n+1}\left(h_{n}\right) \end{array}\right]$$

where *S_N_* is the combined stiffness matrix for the entire structure, $\sigma_{1i}^{0}$, $\sigma_{1i}^{n+1}$are the stress components in the upper and lower bounding layers, respectively, and $u_{i}^{0}\left(0\right)$, $u_{i}^{n+1}\left(h_{n}\right)$ are the displacement components in the upper and lower bounding layers, respectively. The above equation can be solved for the unknown reflection and transmission coefficients. Equation (6) leads to the calculation of 9 unknowns from 6 equations. If the bounding layers are considered to be water, then the above equation is simplified as we know the properties of water, the boundary conditions, and the wave modes supported in water. As water is the upper bounding layer, the incident wave can only be a longitudinal wave, hence we now know the stress and displacement on the top layer caused by the incident wave. Choosing water as the bounding layer reduces the number of unknowns, while also considering the no-slip boundary condition between the upper semi-infinite bounding layer and the first interface, and the lower semi-infinite bounding layer and the last interface,

(7) $$\begin{array}{l} u_{1}^{0}\left(0\right)=u_{2}^{0}\left(0\right)=0\\ u_{1}^{n+1}\left(h_{n}\right)=u_{2}^{n+1}\left(h_{n}\right)=0 \end{array}$$

The reflection coefficient can be calculated using the below equation [18]:

(8) $$R=-\frac{\left(S_{11}^{33}-\Lambda \right)\left(S_{22}^{33}-\Lambda \right)-S_{21}^{33}S_{12}^{33}}{\left(S_{11}^{33}+\Lambda \right)\left(S_{22}^{33}-\Lambda \right)-S_{21}^{33}S_{12}^{33}}$$

where $S_{mn}^{33}$ is the (3,3) component of the constitutive matrices of *S* and

$$\Lambda =-\frac{\cos\theta }{i\omega \rho_{f}V_{f}}$$

where *ρ_f_* and *V_f_* are the density and velocity of sound in water, respectively.

The next section shows the theoretical fundamentals of multi-Gaussian beams.

### 2.2. Modeling of the Transducer Gaussian Beams

The transducer response of phased array at a distance *z* from the face of the transducer can be modeled as a superposition of multi-Gaussian beams [7] as shown below:

(9) $$v_{j}\left(x_{1},\omega \right)=d\exp\left(i\omega \frac{x_{3}}{c}\right)\sum_{m=1}^{10}\sum_{n=1}^{10}\frac{A_{n}A_{m}}{{\sqrt{1+cx_{3}\left[M_{mn}(0)\right]}}_{11}{\sqrt{1+cx_{3}\left[M_{mn}(0)\right]}}_{22}}\exp\left[\frac{1}{2}X^{T}M_{mn}(x_{3})X\right]$$

where ***X*** is the coordinates between the *j*th transmitting element and the receiving elements, *c* is the wave velocity, *x*_3_ is the distance traveled along the *x*_3_ axis in Figure 1, and **d** is the polarization vector.

(10) $$\begin{array}{l} {\left[M_{mn}(0)\right]}_{11}=\frac{iB_{m}}{D_{1}},{\left[M_{mn}(0)\right]}_{22}=\frac{iB_{n}}{D_{2}}\\ D_{1}=\frac{ka_{1}{}^{2}}{2},D_{2}=\frac{ka_{2}{}^{2}}{2} \end{array}$$

(11) $$\begin{array}{l} {\left[M_{mn}(x_{3})\right]}_{11}=\frac{{\left[M_{mn}(0)\right]}_{11}}{1+cx_{3}{\left[M_{mn}(0)\right]}_{11}}\\ {\left[M_{mn}(x_{3})\right]}_{22}=\frac{{\left[M_{mn}(0)\right]}_{22}}{1+cx_{3}{\left[M_{mn}(0)\right]}_{22}}\\ {\left[M_{mn}(x_{3})\right]}_{12}={\left[M_{mn}(x_{3})\right]}_{21}=0 \end{array}$$

In the above equations, *k* is the wavenumber and *a*_1_ and *a*_2_ are the width and length of the rectangular transducer, respectively. *A_n_*, *A_m_*, *B_n_*, and *B_m_* are the Wen and Breazeale coefficients [19]. Wen and Breazeale expressed the radiation from a circular transducer as a superposition of Gaussian beams with coefficients obtained by nonlinear optimization. These coefficients were expanded for a rectangular transducer by Ding et al. [20].

Hence, at the face of the transducer where *x*_3_ = 0 the velocity distribution is given below:

(12) $$v_{j}\left(x,\omega \right)=\sum_{m=1}^{10}\sum_{n=1}^{10}A_{n}A_{m}\exp\left[\frac{1}{2}X^{T}M_{mn}(x_{3})X\right]$$

The velocity distribution in the wavenumber-frequency domain can be calculated as given below:

(13) $$v_{j}\left(k,\omega \right)=\int_{-\infty }^{\infty }v_{j}\left(x,\omega \right)e^{-ikx_{1}}dx_{1}^{}$$

### 2.3. Angular Spectrum of Plane Waves

The method of angular spectrum of waves was first proposed by Goodman [15]. According to the method, finite beam from a transducer can be decomposed using Fourier decomposition into infinite number of plane waves with different angles of propagation in the spatial frequency domain. The angular spectrum method can be carried out by using the Discrete Fourier Transform to transform from wavenumber domain to the spatial domain. The angular decomposition is given by the below equation:

(14) $$F\left(x_{1},x_{2}\right)=\int_{-\infty }^{+\infty }\int_{-\infty }^{+\infty }f\left(k_{x_{1}},k_{x_{2}}\right)e^{-i\left(k_{x_{1}}x_{1}+k_{x_{2}}x_{2}+k_{x_{3}}x_{3}\right)}dk_{x_{1}}dk_{x_{2}}$$

where $f\left(k_{x_{1}},k_{x_{2}}\right)$ is the acoustic wave field at the face of the transducer in the wavenumber domain and $F\left(x_{1},x_{2}\right)$ is the acoustic wavefield in the spatial domain. $k_{x_{1}}$and $k_{x_{2}}$are the wavenumber components in the plane normal to the plane of propagation of the wave. For 2-D inspection, the wavenumber in the *y* direction can be considered as 0, and Equation (14) simplifies to the equation given below:

(15) $$F\left(x_{1},0\right)=\int_{-\infty }^{+\infty }f\left(k_{x_{1}},0\right)e^{-i\left(k_{x_{1}}x_{1}+k_{x_{3}}x_{3}\right)}dk_{x_{1}}$$

### 2.4. Modeling of Array Signals to Simulate FMC

For modeling the array signals from a Gaussian beam transducer, we combine the multi-Gaussian beam model for the transducer elements with the response of the layered material using the stiffness matrix approach as given below:

(16) $$F\left(x_{1},0\right)=\int_{-\infty }^{+\infty }v_{j}\left(k_{x_{1}},0\right)\beta \left(\omega \right)R\left(k_{x_{1}},0\right)e^{-i\left(k_{x_{1}}x_{1}+k_{x3}x_{3}\right)}dk_{x_{1}}$$

where β(ω) is the combined system function for a pair of transducer and receiver elements, $R\left(k_{x_{1}},0\right)$ is the reflection coefficient of the entire structure calculated using Equation (8), and *x*_1_ is the distance between *j*th element and the receiver position.

The system function for an array element can be calculated in the following way as proposed by Schmerr [21]:

1. The backwall echo response $F_{BWE}$ for a transducer element from a known material such as aluminium, etc. is calculated experimentally.
2. The backwall echo $F_{BWA}$ is then calculated analytically using a simple testing configuration.

It is assumed that the relationship between the experimental and analytical backwall echo is given by Equation (17)

(17) $$F_{BWE}=\beta \left(\omega \right)F_{BWA}$$

Hence, the combined system response between a pair of elements is given below:

(18) $$\beta \left(\omega \right)=F_{BWE}/F_{BWA}$$

The deconvolution process in Equation (18) is carried out by implementing a Weiner Filter to reduce the sensitivity to noise as given below [22]:

$$\beta \left(\omega \right)=\frac{F_{BWE}\left(\omega \right)F_{BWA}{}^{*}\left(\omega \right)}{\left[|F_{BWA}{|}^{2}+\varepsilon^{2}\max\left\{|F_{BWA}{|}^{2}\right\}\right]}$$

where ^*^ refers to the complex conjugate, and ε is a small noise constant.

It is assumed that the elements are linear, time-invariant, and identical in frequency response and directivity as those demonstrated by Huang [7]. Hence, the combined system response for just one pair of elements is required to characterize the other elements.

## 3. Simulation and Experimental Results

In this section, simulation and experimental results will be presented. The calculations are carried out using three transducer arrays of different center frequencies, array size, and number of elements.

The experimental FMC signals were acquired using the FI ToolBox from Diagnostic Sonar. The transducers used were phased array transducers supplied by Olympus© (Leiderdorp, Netherlands). The signals were captured using the Diagnostic Toolbox and were imported into MATLAB^®^ for plotting the data. The simulations were carried out using MATLAB 2017^®^.

**Figure 2:**
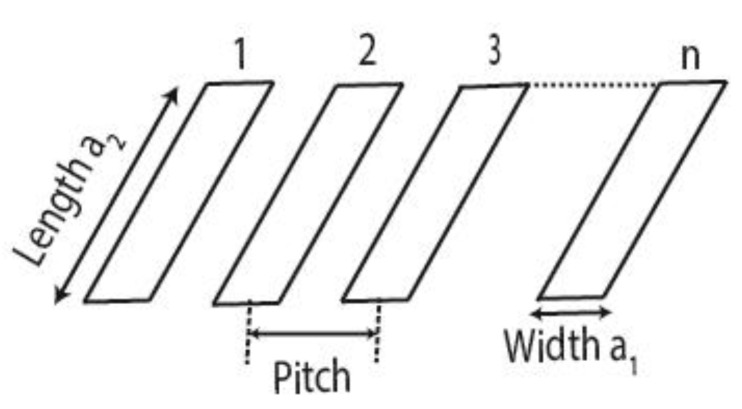
Elements of an array transducer.

Figure 2 shows the transducer array configuration used for the experiments and simulations, where 1, 2 donate the array element number and *n* is the total number of array elements.

The specifications of the transducers are shown in Table 1.

For simulation and experimental purposes, we consider an aluminum block 25 mm thick and a CFRP laminate, which is quasi-isotropic and 19 mm thick with (0/45/−45/90) layup. There are 169 layers of UniDirectional CFRP prepreg of 110 μm thickness in the laminate with layer of epoxy resin of thickness 5 μm between them. The properties of aluminum and unidirectional CFRP lamina [23] are given in Table 2.

By taking the complex material properties, we take into account the attenuation caused due to viscoelasticity in the CFRP lamina. The simulation consists of evaluating the Equations (8), (13) and (18) and then substituting the results in Equation (16).

### 3.1. Total Reflection Coefficient of the Materials under Inspection

**Figure 3:**
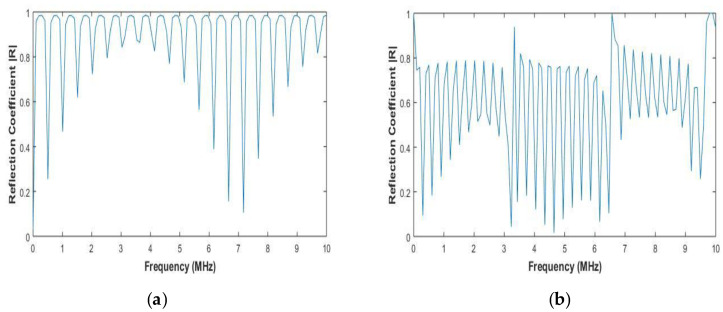
(**a**) Total reflection coefficient for aluminum (**b**) Total reflection coefficient for CFRP laminate.

The reflection coefficient was obtained by evaluating Equation (8) for the materials in Table 1 at different frequencies and for normal incidence. The reflection coefficient is given in Figure 3a,b.

Figure 3a,b shows the reflection coefficients for aluminium and CFRP, respectively. The plane wave reflection coefficient is frequency-dependent, which can be observed in Figure 3. The resonance is characterized by a reflection coefficient of 1, which is observed in Figure 3. At the resonant frequencies, the transmission coefficient is 0. The resonant frequencies can also be analytically calculated by the below equation:

(19) $$RF=\frac{nc}{2d}$$

where *n =* 1, 2, 3, *…*, *c* is the velocity of the ultrasonic wave in the material, and *d* is the total thickness of the material. The resonant frequencies are dependent on the thickness of the material system and velocity of the wave in it. The resonant frequencies calculated using Equation (19) are equal to the resonant frequencies observed by evaluation of Equation (8). Therefore, the reflection coefficients at various frequencies and wavenumbers can be calculated using the stiffness matrix method and substituted in Equation (16).

### 3.2. System Functions of the Transducer Arrays

**Figure 4:**
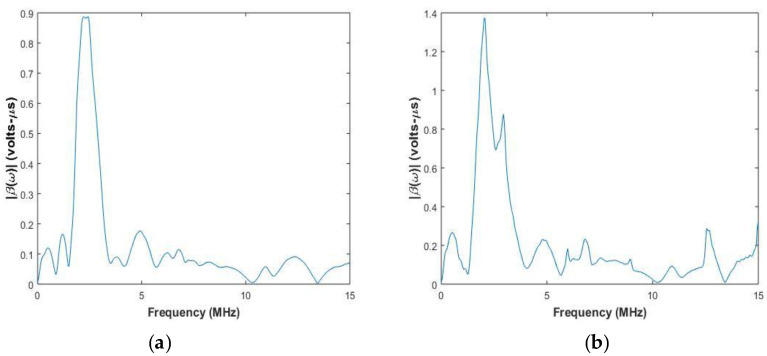
System function of pair of elements with center frequency of 2.25 MHz used for testing (**a**) Aluminium (**b**) CFRP laminate.

**Figure 5:**
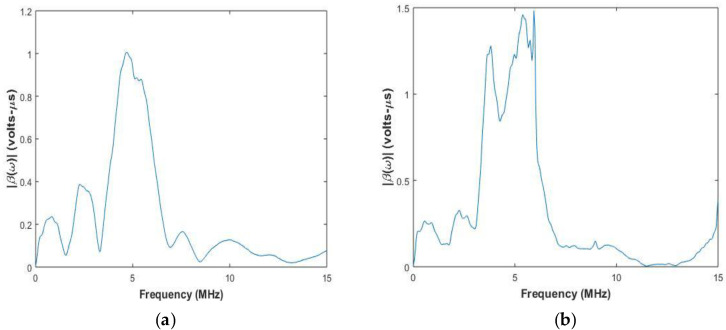
System function of pair of elements with center frequency of 5 MHz used for testing (**a**) Aluminium (**b**) CFRP laminate.

The system function is calculated by using Equation (18). Figure 4 and Figure 5 show the system functions for elements of center frequency 2.25 MHz and 5 MHz used for inspection of the aluminum block and the CFRP laminate.

In Figure 4 and Figure 5, it can be observed that the system function peak is at the center frequency of the transducer, and the width of the peak depends on the transducer bandwidth. For CFRP laminate in Figure 4b, it is observed that there is another peak closer to the central peak. This is attributed to the surface of the CFRP laminate under inspection, which can also affect the system function. The system function has to be calculated whenever there is a change in central frequency of the transducer or the material under inspection.

### 3.3. Comparison of Experimental and Simulated FMC Signals

**Figure 6:**
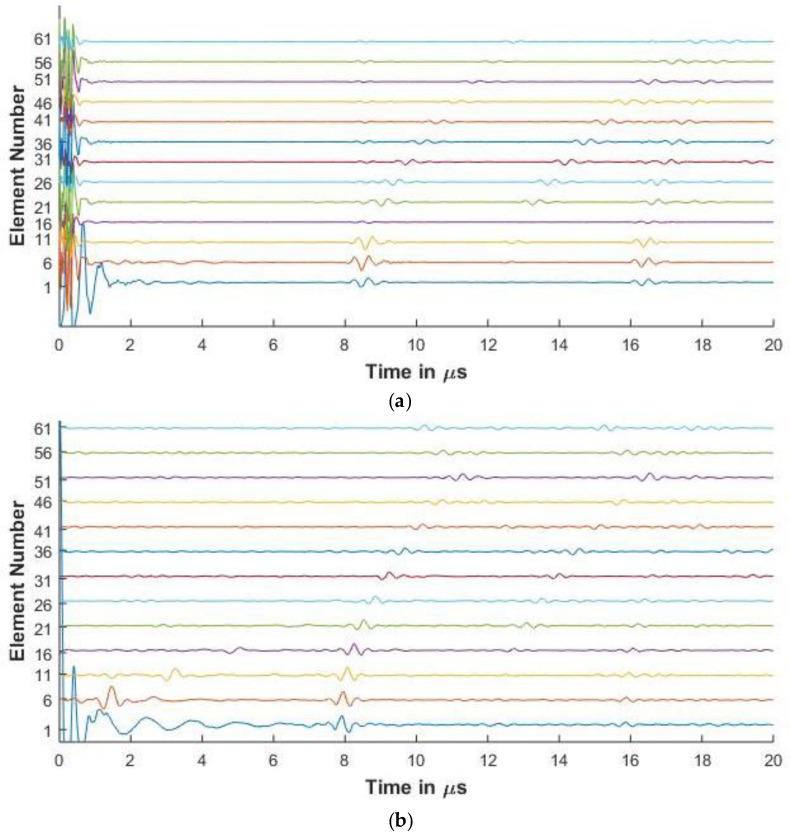
(**a**) Experimental full matrix capture (FMC) signals obtained for aluminium with 2.25 MHz 64 element array (**b**) Simulated FMC signals for aluminium with 2.25 MHz 64 element array.

**Figure 7:**
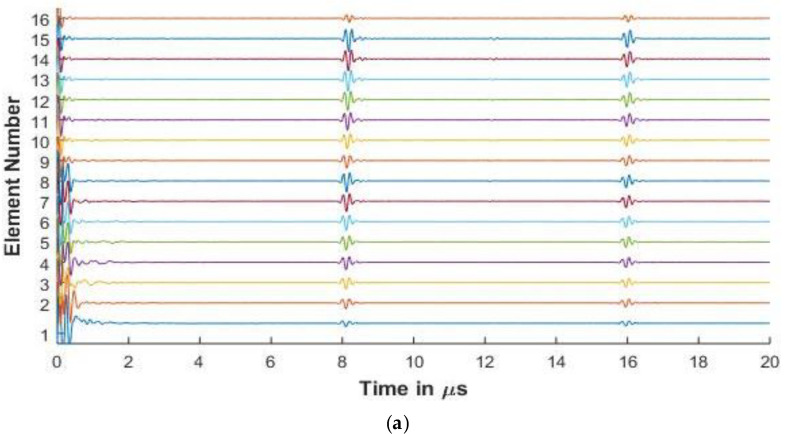

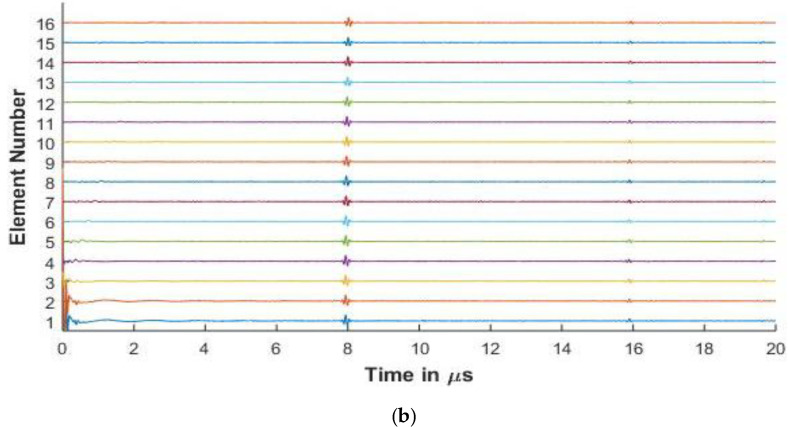
(**a**) Experimental FMC signals obtained for aluminum with 5 MHz 16 element array (**b**) Simulated FMC signals for aluminum with 5 MHz 16 element array.

**Figure 8:**
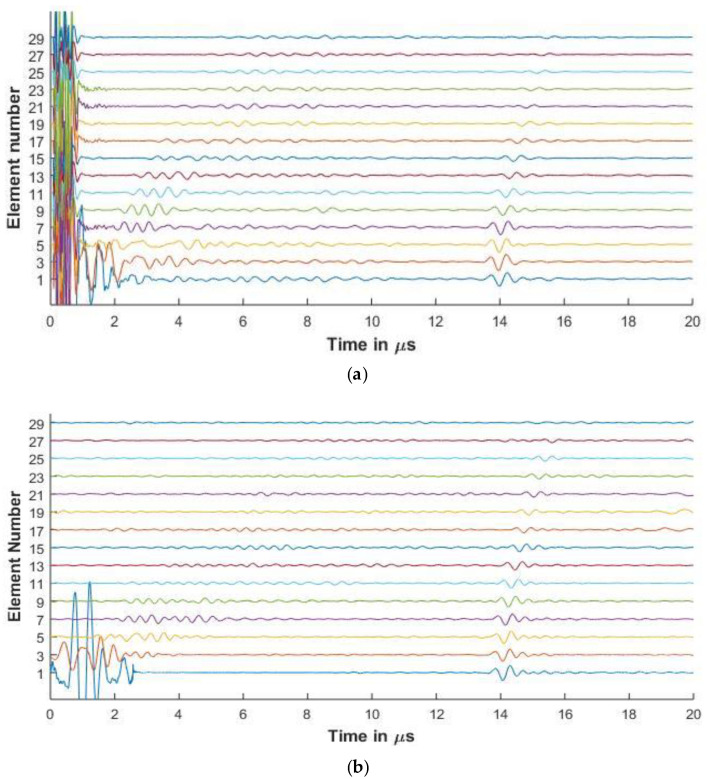
(**a**) Experimental FMC signals obtained for CFRP with 2.25 MHz 64 element array (**b**) Simulated FMC signals for CFRP with 2.25 MHz 64 element array.

**Figure 9:**
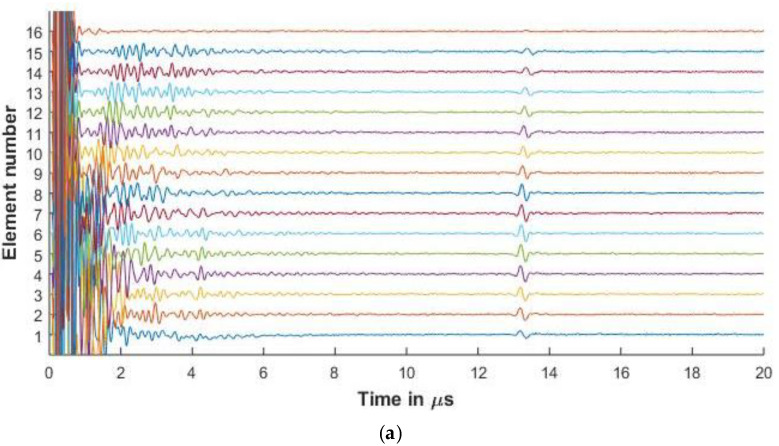

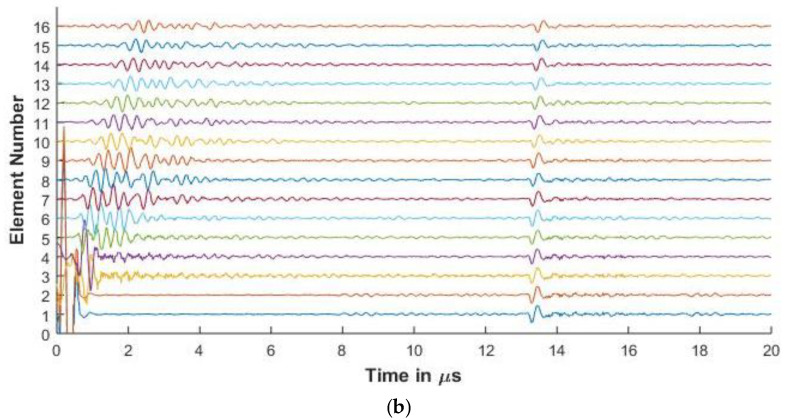
(**a**) Experimental FMC signals obtained for CFRP with 5 MHz 16 element array (**b**) Simulated FMC signals for CFRP with 5 MHz 16 element array.

**Figure 10:**
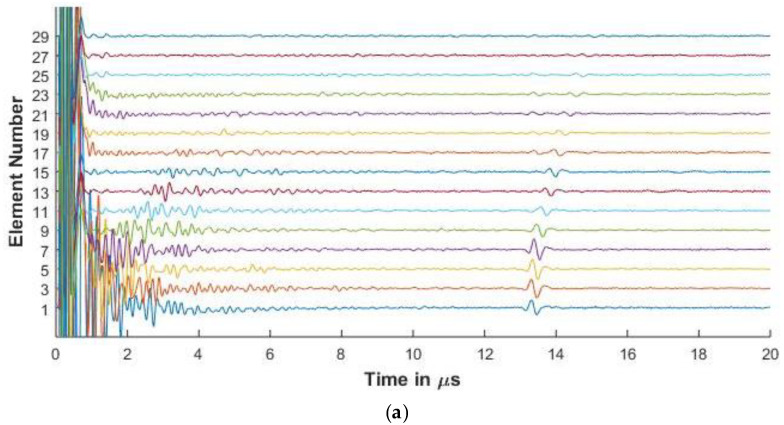

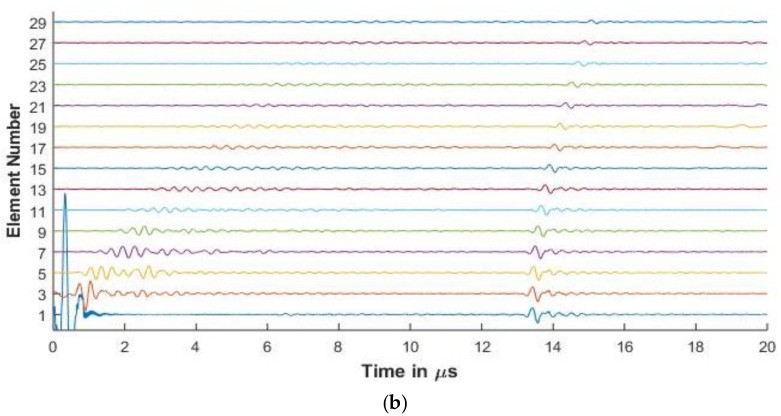
(**a**) Experimental FMC signals obtained for CFRP with 5 MHz 128 element array (**b**) Simulated FMC signals for CFRP with 5 MHz 128 element array.

For the purposes of this paper, in Figure 6, Figure 7, Figure 8, Figure 9 and Figure 10, the 1st element, as shown in Figure 2, is the transmitting element while the others are receiving elements. Similar figures can be plotted for the other transmitting and receiving elements from the simulated FMC data.

#### 3.3.1. Experimental and Simulated FMC Signals in Aluminum

Figure 6 and Figure 7 present the experimental and simulated FMC signals for inspection of aluminum with Arrays 1 and 2, respectively. In Figure 6 and Figure 7, the backwall echo can be clearly seen between 8 and 10 μs. The simulated signals agree with the experimentally determined signals. The front surface reflected signal can be observed at various elements. The slower quasi-shear waves can be seen between 12 and 16 μs. The second backwall echo is observed at 17 μs. The signals decrease in amplitude as we move away from the firing element due to material attenuation and the effects of diffraction.

In Figure 6a, a small signal is observed just before 8 μs. This signal is seen in the experimental result and is missing in the simulation. The signal is a relatively low amplitude signal that can be attributed to small inconsistencies in the experimental aluminum reference block provided by Olympus© (Leiderdorp, Netherlands). In Figure 6b, a low amplitude signal can be seen in the simulation results before the backwall echo for elements 56 and 61. These are attributed to the noise signals generated while synthesizing the simulated signals from a high sampling rate, which is done so as to correspond with the Nyquist frequency of 50 MHz of the experimental results. 

Table 3 presents the comparison of the first element backwall amplitude reduction between experimental and simulated results

It can be observed from the table that the difference between the reduced amplitude between the simulated and the experimental results is less than 2 dB, showing good agreement between the experimental and simulated results. The percentage error between the experimental and simulated results for the 2.25 MHz and 5 MHz is calculated to be 1.89% and 3.63%, respectively, showing agreement between the simulation and experimental results.

#### 3.3.2. Experimental and Simulated Signals in CFRP

Figure 8 and Figure 9 show the experimental and simulated signals for the CFRP laminate. The amplitude of the backwall echoes is reduced as compared to aluminium due to the increased attenuation of signals. The reduction of the signals is due to attenuation caused by viscoelasticity of the lamina, diffraction effects, and the scattering of the wave from the ply interfaces. The signals presented for the 64 and 128 elements array are up to element 30 as the elements beyond this do not receive the reflected signal owing to the losses as stated above. 

Ply resonances can also be observed in the signals. It is also seen that as the center frequency of the signal increases, the amplitude of the ply resonances and also the scattering from the interfaces increases as seen in Figure 8 and Figure 9. The slower shear waves cannot be observed due to the increased attenuation of the laminate.

In Figure 8a for elements 1, 3, and 5, signals are observed that occur before the backwall echo. These are missing from the simulated results. This can be attributed to the fact that although the simulation takes the layer attenuation and reflections into consideration, the layers in the manufactured material are not of equal thicknesses and might have pockets of resin and other small defects that influence the signal.

Further in the simulated signals of Figure 8b and Figure 10b, low amplitude signals can be observed at 20 μs. These low amplitude signals arise as the sampling rate is high to avoid aliasing and hence leads to the computation of a large number of frequencies. Due to this, when the inverse Fourier transform is carried out, the time window is longer than 20 μs and contains noise generated due to large number of sampling frequencies. As the output signals are cut at 20 μs for comparison with the experimental results, some of these low amplitude noisy signals are seen.

As a comparison of the effect of the element pitch on the FMC signals, Array 3 is used to inspect the CFRP laminate. In Figure 10, the increased pitch and element width of Array 3 show less resonance from the plies as compared to Figure 9. This shows one of the ways the simulation model can be used to optimize the array parameters for different thickness and material properties.

It can be observed that the experimental front wall echo consists of noise and hence is not suitable for comparison between the experimental and simulated results. Hence, Table 4 presents the difference of 1st and 3rd element backwall amplitude between experimental and simulated results.

It can be observed from the table that the difference between the reduced amplitude between the simulated and the experimental results is less than 1 dB, showing good agreement between the results. The percentage error between the experimental and simulated results for the 2.25 MHz and 5 MHz is calculated to be 5.5% and 1.43%, respectively, showing agreement between the simulation and experimental results.

## 4. Discussions

As shown in Figure 3, the stiffness matrix method predicts the reflection coefficient and the resonant frequency of the system under inspection. This can help in determining the resonant frequency of the material under inspection and hence lead to a better choice of inspection as at resonant frequencies the wave undergoes total reflection, leading to no penetration of the material. 

Figure 6, Figure 7, Figure 8 and Figure 9 show that proposed modeling techniques to simulate the received FMC signals are in good agreement to the experimental results as also shown in Table 3 and Table 4 with percentage errors less than 6% between the results. Slight discrepancies are noted in the experimental and simulated results. As discussed, these discrepancies arise due to the different ultrasonic velocities used in the simulation and in the experimental acquisition owing to slightly different material properties of the experimental and simulated samples. The low amplitude noise is also present in the simulated results which is due to the signal being synthesized from a large number of frequencies. 

The thickness of the couplant used and its properties also influence the time of flight as in the simulation the laminate is surrounded by water bounding layers of infinite thickness, whereas in the experimental scenario the couplant gel layer has a finite thickness and slightly different wave velocity. As discussed in Section 2, water bounding layers are assumed due to the fact that only longitudinal waves can travel through water, hence only incident longitudinal waves need to be considered entering the material under inspection. Due to this, the number of unknowns reduces in Equation (6), enabling us to solve 6 equations for 6 unknowns.

The extensive electrical response of array elements is missing in the simulated results, but the computation of the system response by using the backwall method captures the other saliant frequency response of the array elements. The frequency response is observed to be not precisely Gaussian in shape due to different varying factors such as the electronic components of the setup, the top surface of the material inspected, thickness of couplant, etc. 

The trend of the diminishing backwall echos as we move further away from the transmitting element is identical for both the experimental and the simulated results. In Figure 8, Figure 9 and Figure 10, we can observe the reverberations from the layers in the simulated results, which agree with the experimental results. These reverberations tend to contribute to the noise of the signal and hence the ability of the modeling technique to simulate these for the material under inspection can help in optimizing the array parameters without extensive experimental analysis. The slight discrepancy in the results of the CFRP laminate can be attributed to the slightly varying thickness of layers and resin in the manufactured laminate as in the simulation it is assumed that the layers are of constant thickness and parallel to each other. Figure 9 and Figure 10 also provide a comparison between the array signals received due to different element sizes and pitch, which influence the received FMC signals.

## 5. Conclusions

The paper proposes a Gaussian beam and recursive stiffness matrix-based modeling techniques to model the FMC signals from layered CFRP laminates. The simulated signals have good agreement, to within 2 dB and a percentage error of less than 6% with the experimental signals, and are able to simulate the different components of the experimental signal, which include ply resonances, front wall reflection, backwall echos, and also the backwall echos from the slower shear waves. The proposed model takes into account the diffraction effects caused by Gaussian beams and mimics the real-world scenario where the transducer emits Gaussian-shaped beams. It is also shown how the model can be used to optimize various parameters of the inspection process. The model can be used for both isotropic and anisotropic layered media, where the anisotropic group velocity is taken into account.

## Figures and Tables

**Table 1:** Transducer array specifications.

	Centre Frequency (MHz)	Pitch (mm)	Number of Elements
Array 1	2.25	1	64
Array 2	5	0.6	16
Array 3	5	1	128

**Table 2:** Material properties [23].

Properties	Aluminum (GPa)	Carbon/Epoxy >65% Fibre-Volume Fraction (GPa)
C_11_	110	13.89 (1 + 0.02i)
C_22_	110	13.89 (1 + 0.02i)
C_33_	110	121.7 (1 + 0.001i)
C_12_ = C_21_	60	6.43 (1 + 0.011i)
C_13_ = C_31_	60	5.5 (1 + 0.007i)
C_23_ = C_32_	60	5.5 (1 + 0.007i)
C_44_	25	5.1 (1 + 0.066i)
C_55_	25	5.1 (1 + 0.066i)
C_66_	25	3.73 (1 + 0.027i)

**Table 3:** Comparison of backwall amplitude reduction.

Frequency (MHz)	Experimental	Simulation
2.25	40.16 dB	39.4 dB
5	10.87 dB	11.28 dB

**Table 4:** Comparison of backwall echo difference between experimental and simulated results.

Frequency	Experimental	Simulation
2.25	5.82 dB	5.5 dB 5.5%
5	6.9 dB	7 dB 1.43%
